# Circ_0005198 enhances temozolomide resistance of glioma cells through miR-198/TRIM14 axis

**DOI:** 10.18632/aging.202234

**Published:** 2020-12-09

**Authors:** Yanyao Deng, Hongwei Zhu, Le Xiao, Chao Liu, Xiangrui Meng

**Affiliations:** 1 Department of Neurology, The First Hospital of Changsha, Changsha 410005, Hunan, China; 2 Department of Hepatopancreatobiliary Surgery, The Third Xiangya Hospital, Central South University, Changsha 410013, Hunan, China; 3 Department of Gastroenterology, The Third Xiangya Hospital, Central South University, Changsha 410013, Hunan, China

**Keywords:** circ_0005198, miR-198, TRIM14, TMZ resistance, glioma

## Abstract

Circular RNAs (circRNAs) are associated with chemoresistance in many cancers. However, the function of circ_0005198 in the temozolomide (TMZ) resistance of glioma has not been well elucidated. Here, we demonstrated that circ_0005198 was considerably up-regulated in glioma tissues, serum samples and TMZ-resistant glioma cells. Silencing of circ_0005198 restrained TMZ resistance, restricted the proliferation and facilitated the apoptosis of TMZ-resistant glioma cells. MiR-198 could be sponged by circ_0005198, and we demonstrated that the effect of circ_0005198 on the progression of TMZ-resistant glioma cells was attributed to the inhibition of miR-198 activity. Moreover, TRIM14 was a target of miR-198 and silencing of TRIM14 hindered TMZ resistance and suppressed the progression of TMZ-resistant glioma cells, while TRIM14 over-expression rescued the inhibiting effect of miR-198 over-expression. We conclude that circ_0005198-miR-198-TRIM14 regulatory pathway is critical to TMZ resistance of glioma.

## INTRODUCTION

Glioma originates from the neuroglial stem or progenitor cells and is a malignancy with strong aggressiveness [1]. With the advancement of surgery-related technologies and neoadjuvant chemical therapy, the mortality rate of glioma has considerably decreased, and the prognosis has improved [2]. Unfortunately, prognosis remains poor for patients enhancing resistance to chemotherapeutic drugs. Increasing evidence has shown that glioma stem cells (GSCs) account for tumor proliferation even after chemotherapy [3]. For this reason, delving into the molecular pathogenesis of glioma and the mechanism of chemoresistance acquisition will facilitate the advancement of personalized medicine as well as targeted therapies.

Circular RNAs (circRNAs) refer to one type of endogenous non-coding RNAs and present in the form of closed loops which cannot code protein [4]. Existing researches reported that circRNAs participate in a wide variety of physiological and pathophysiological procedures (e.g., concocting modulating alternative splicing, sponging microRNA (miRNA), as well as regulating the interactions and genetic expression of protein-RNA) [5–7]. Moreover, abnormally expressed circRNAs are implicated with the progression of various tumors [8, 9]. For instance, circ_001680 could facilitate colorectal carcinoma proliferation and migration and mediate its chemoresistance [10]. Moreover, circRBM33 could regulate IL-6 to facilitate gastric cancer progression [11]. Circ_0005198 was mapped to chr13:25072253-25077915 with a spliced length of 592 nt and involved in the regulation of invading, migrating, apoptotic, and proliferating processes in glioma [12]. However, its effect in the chemoresistance of glioma is still worth further exploration.

Recent data has also demonstrated the key effect of circRNAs in the development of cancers by sponging microRNAs and regulating RNA binding proteins [13, 14]. We have predicted that miR-198 has putative binding sites of circ_0005198, based on our search in Circular RNA Interactome (https://circinteractome.nia.nih.gov/index.html). MiR-198 was verified involved in the progression of malignant melanoma [15]. Also, Liu et al. reported that miR-198 could inhibit the development of papillary thyroid carcinoma [16]. Nevertheless, the role of miR-198 in regulating the biological processes of glioma is largely unexplored.

Tripartite motif-containing 14 (TRIM14), consisting of a B-box, a coiled-coil region and a C-terminal PRYSPRY region, involves in cell apoptosis, cell cycle regulation and viral response [17]. We speculated that TRIM14 is the putative target of miR-198 based on the website database TargetScan (http://www.targetscan.org/). Previous studies demonstrated that TRIM14 could be targeted by miR-124-3p to impact tongue cancer development [18]. Besides, TRIM14 facilitated epithelial-mesenchymal transition and chemoresistance of glioma [19, 20]. Hence, TRIM14 was expected to be a good target for glioma treatment.

The aim of our study here was to investigate the role of circ_0005198 in TMZ resistance of glioma cells and dissect its mechanism via bioinformatics prediction and experimental verification. The discovery of the circ_0005198-miR-198-TRIM14 axis is promising to provide a novel therapeutic insight for glioma chemoresistance.

## RESULTS

### Circ_0005198 expression was increased in glioma

**Figure 1:**
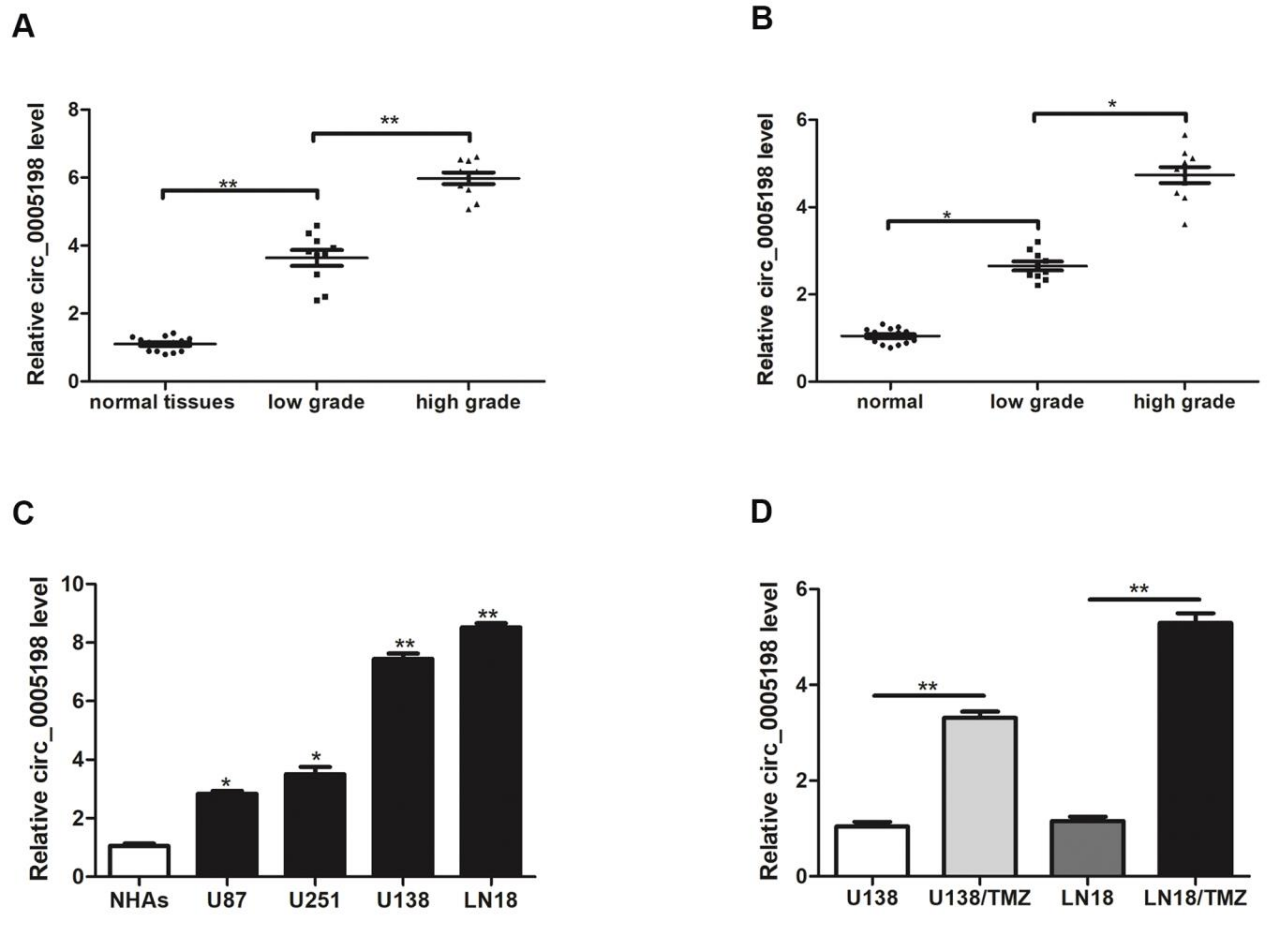
**Circ_0005198 expression was upregulated in glioma tissues, serum samples and cells.** (**A**) qRT-PCR was used to measure the expression of circ_0005198 in 10 low-grade (stage l-ll) glioma tissues, 10 high-grade (stage lll-IV) glioma tissues and 15 normal tissues**.** (**B**) The expression of circ_0005198 in serum was assessed by qRT-PCR. (**C**) Circ_0005198 expression was detected by qRT-PCR in normal human astrocytes (NHAs) and glioma cell lines (U87, U251, U138 and LN18). (**D**) The expression of circ_0005198 in glioma cells (U138 and LN18) and TMZ-resistant glioma cells (U138/TMZ and LN18/TMZ) was assessed by qRT-PCR. *P < 0.05, **P< 0.01.

Firstly, we tested the expression of circ_0005198 in glioma. In clinical samples, the expression level of circ_0005198 was higher in glioma tissues than normal tissues and its expression displayed positive correlations with tumor grades (Figure 1A). We then examined the expression of circ_0005198 in serum samples. As shown in Figure 1B, circ_0005198 expression was gradually increased in glioma compared to the control, and its expression also displayed positive correlations with tumor grades. Besides, we showed that the expression of circ_0005198 was up-regulated in all glioma cell lines *in vitro*, especially in U138 and LN18 cells, compared to normal human astrocytes (Figure 1C). Furthermore, circ_0005198 expression in U138/TMZ and LN18/TMZ cells was higher than that in U138 and LN18 cells (Figure 1D), demonstrating that circ_0005198 might be related to TMZ resistance of glioma.

### Circ_0005198 knockdown inhibited the progression of TMZ-resistant glioma cells

**Figure 2:**
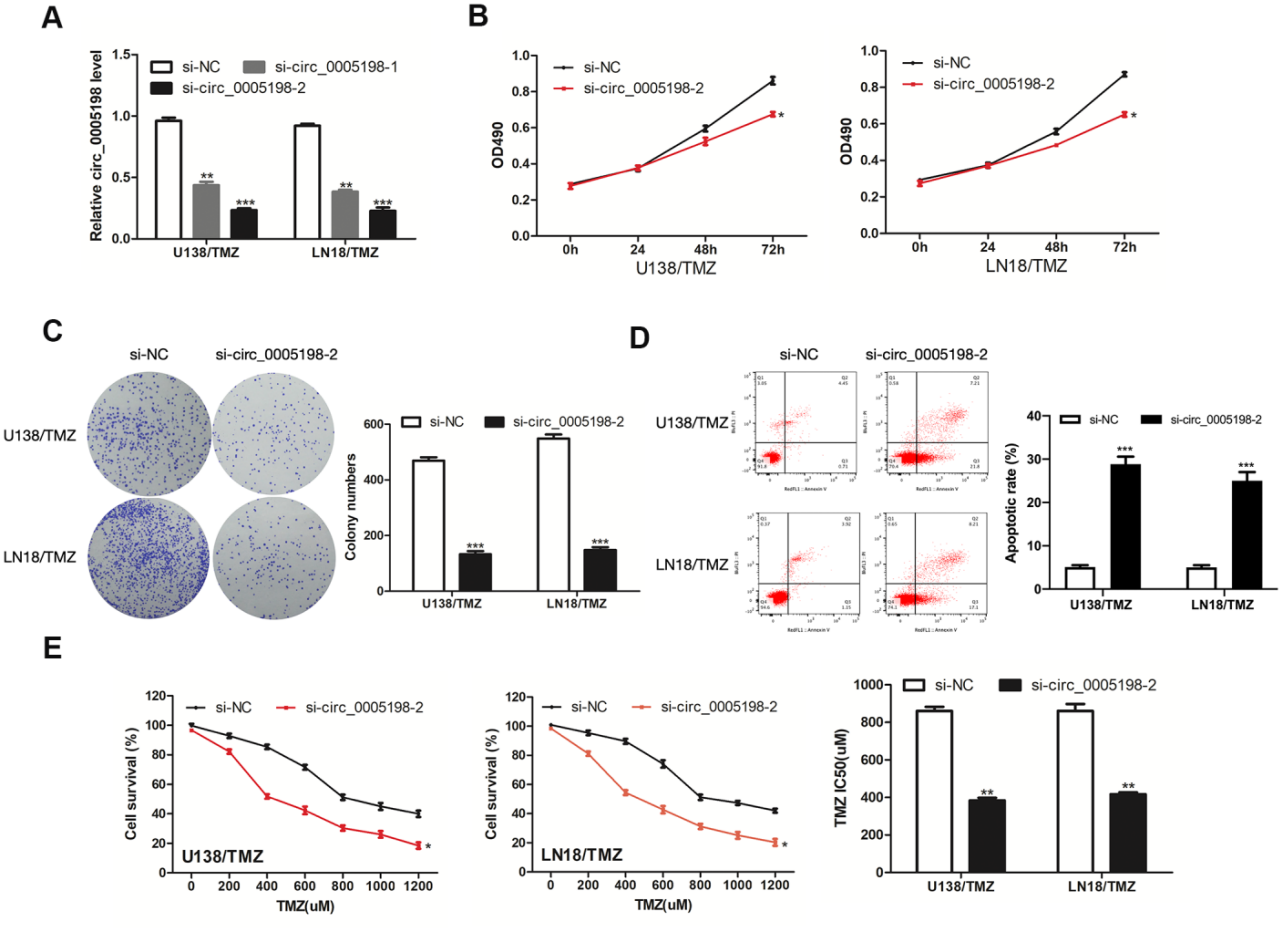
**Effects of circ_0005198 expression on the progression of TMZ-resistant glioma cells. (A)** qRT- PCR analysis of circ_0005198 expression in transfected U138/TMZ and LN18/TMZ cells. (**B**) The viability of U138/TMZ and LN18/TMZ cells transfected with si-circ_0005198-2 were detected by CCK-8 assay. (**C**) The proliferative rate of transfected U138/TMZ and LN18/TMZ cells was determined by colony formation assay. (**D**) The apoptotic rate of cells was determined using flow cytometry after circ_0005198 knockdown in U138/TMZ and LN18/TMZ cells. (**E**) CCK-8 assay was performed to measure the TMZ resistance of U138/TMZ and LN18/ TMZ cells. *P <0.05, **P < 0.01, *‘*P < 0.001.

Circ_0005198 expression was knocked down in U138/TMZ and LN18/TMZ cells to investigate the biological function of circ_0005198 in glioma cells (Figure 2A). Cell viability assay showed that the cell proliferation of U138/TMZ and LN18/TMZ cells was significantly inhibited by circ_0005198 knockdown (Figure 2B). Likewise, colony formation assays showed that the clone numbers of U138/TMZ and LN18/TMZ cells were significantly reduced with circ_0005198 down-regulation as well (Figure 2C). The apoptotic rate of U138/TMZ and LN18/TMZ cells transfected with si-circ_0005198-2 was increased (Figure 2D) compared to the scrambled group. The IC50 value of cells was significantly decreased after circ_0005198 knockdown, indicating that TMZ resistances of U138/TMZ and LN18/TMZ cells were suppressed (Figure 2E). As a result, we verified that circ_0005198 played an active effect in TMZ resistance of glioma cells.

### Circ _0005198 directly interacted with miR-198

**Figure 3:**
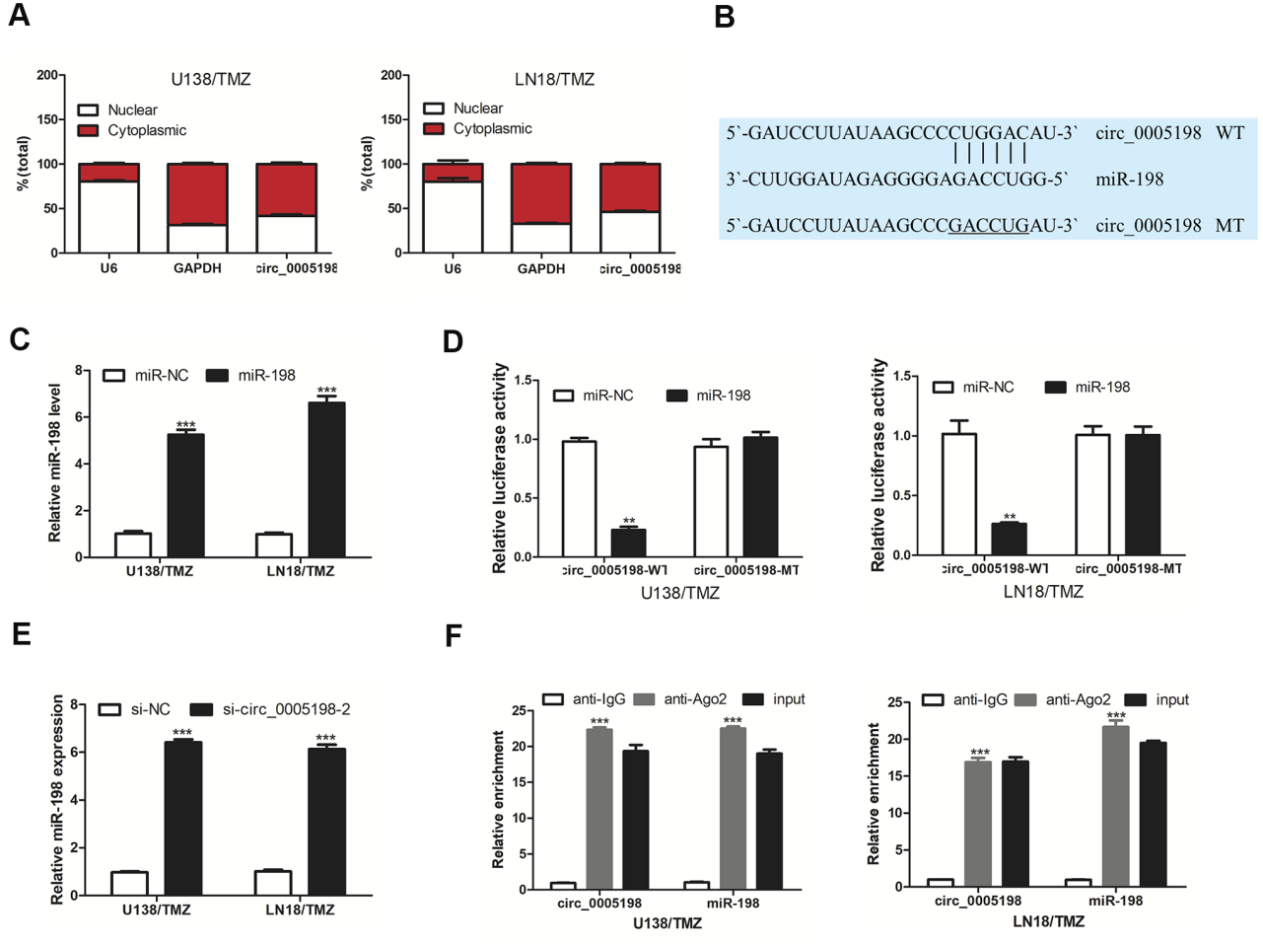
**Circ 0005198 targeted miR-198.** (**A**) qRT-PCR showed the enrichment of circ_0005198 in cytoplasmic. (**B**) The predicted binding sites between circ_0005198 and miR-198. (**C**) MiR-198 expression in U138/TMZ and LN18/TMZ cells was detected by qRT-PCR after transfection with miR-138 mimic. (**D**) Luciferase reporter assay was performed for the confirmation of direct binding relationship between circ_0005198 and miR-198 with luciferase reporter plasmids of wild-type and mutant circ_0005198. (**E**) qRT-PCR was performed to detect the effect of circ_0005198 on miR-198 expression. (**F**) Enrichment of circ_0005198 and miR-198 using Ago2 antibody in U138/TMZ and LN18/TMZ cells was tested by RIP assay. **P < 0.01, ***P < 0.001.

Subsequently, we attempted to delve into the possible action mechanism of circ_0005198 in glioma cells. Predominantly cytoplasmic enrichment of circ_0005198 was identified (Figure 3A), demonstrating that circ_0005198 might hinder target mRNA repression as the molecular sponges for miRNAs. Based on Circular RNA Interactome, miR-198 binding sequence was shared by that of circ_0005198 at 3′-UTR (Figure 3B). We transfected miR-198 mimic into U138/TMZ and LN18/TMZ cells respectively (Figure 3C). Over-expression of miR-198 inhibited luciferase activity in U138/TMZ and LN18/TMZ cells transfected with circ_0005198-WT but not in cells transfected with circ_0005198-MT (Figure 3D). In particular, miR-198 expression level was elevated considerably with circ_0005198 knockdown (Figure 3E). Furthermore, RIP assay proved the specific interaction of circ_0005198 and miR-198 in U138/TMZ and LN18/TMZ cells (Figure 3F). Taken together, circ_0005198 could act as a sponge for miR-198.

### Over-expression of miR-198 inhibited the progression of TMZ-resistant glioma cells

**Figure 4:**
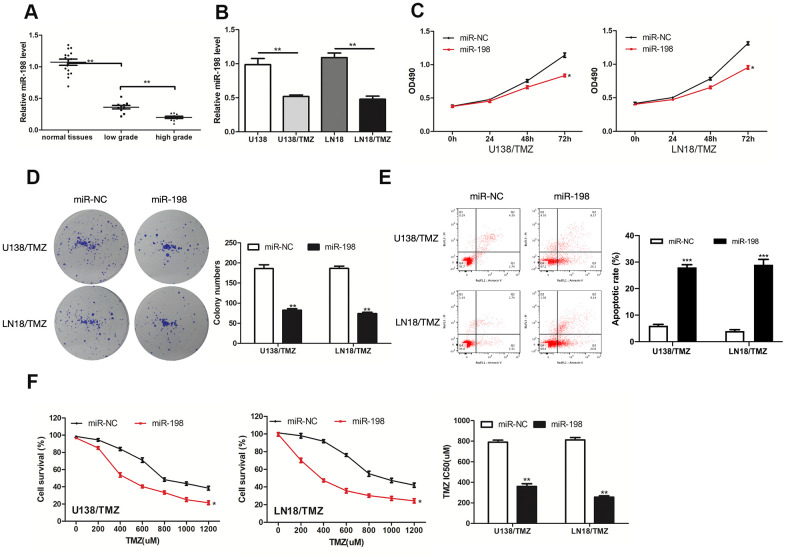
**MiR-198 was down-expressed in glioma and its overexpression inhibited the progression of TMZ-resistant glioma cells.** (**A, B**) Analysis of the relative expression of miR-198 in glioma tissues, glioma cells (U138 and LN18) and TMZ-resistant glioma cells (U138/TMZ and LN18/TMZ). (**C**) The viability of U138/TMZ and LN18/TMZ cells transfected with miR-198 mimic was detected by CCK-8 assay. (**D**) Proliferation of U138/TMZ and LN18/TMZ cells was determined by colony formation assays. (**E**) Apoptotic rate in U138/TMZ and LN18/ TMZ cells was detected by flow cytometry. (**F**) The TMZ resistance of U138/TMZ and LN18/TMZ cells was measured by CCK-8 assay. *P < 0.05, **P < 0.01, ***P < 0.001.

Next, the impacts exerted by miR-198 on TMZ resistance of glioma were explored. MiR-198 was down-regulated in glioma tissues and TMZ-resistant glioma cells (Figure 4A, 4B). We further elevated the expression of miR-198 in TMZ-resistant glioma cells by transfecting miR-198 mimic. As revealed from the results of CCK-8 and colony formation assays, miR-198 over-expression restricted U138/TMZ and LN18/TMZ cells proliferation (Figure 4C, 4D). The apoptotic rates of U138/TMZ and LN18/TMZ cells transfected with miR-198 mimic were increased (Figure 4E). The IC50 value of the cells evidently declined after miR-198 over-expression, indicating that TMZ resistance was suppressed (Figure 4F). Given the mentioned data, miR-198 was critical to the TMZ resistance of glioma.

### Circ_0005198 was mediated by the negative regulation of miR-198

**Figure 5:**
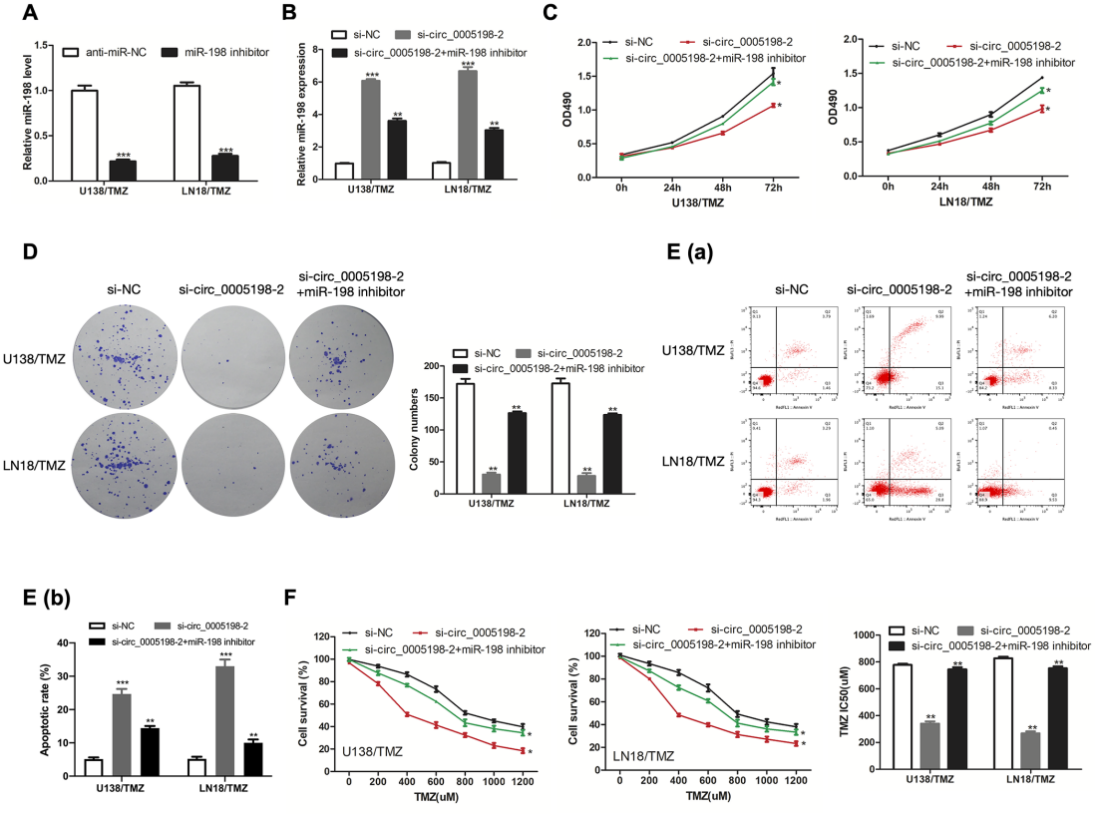
**Circ_0005198 functioned via negatively regulating miR-198 expression.** (**A**) MiR-198 expression in U138/TMZ and LN 18/TMZ cells was detected by qRT-PCR after transfection with miR-138 inhibitor. (**B**) qRTPCR was performed to detect miR-198 expression in U138/TMZ and LN18/TMZ cells after co-transfected with sicirc_0005198-2 and miR-198 inhibitor. (**C**, **D**) The viability of U138/TMZ and LN18/TMZ cells co-transfected with si-circ_0005198-2 and miR-198 inhibitor was detected by CCK-8 and colony formation assays. (**E**) Apoptotic rate in U138/TMZ and LN18/TMZ cells was detected by flow cytometry. (**F**) The TMZ resistance of U138/TMZ and LN18/TMZ cells was measured by CCK-8 assay. *P < 0.05, **P < 0.01, ***P < 0.001.

We then inhibit miR-198 level by transfecting miR-198 inhibitor into U138/TMZ and LN18/TMZ cells (Figure 5A). MiR-198 expression was enriched by si-circ_0005198-2 and partially reversed via co-transfection with miR-198 inhibitor (Figure 5B). As indicated from the results of CCK-8 and colony formation assays, the cell proliferation inhibition mediated by si-circ_0005198-2 could receive partial reversion by co-transfection with miR-198 inhibitor (Figure 5C, 5D), demonstrating that circ_0005198 facilitated cell proliferation by restricting miR-198 expression. Additionally, flow cytometry assay showed that the apoptotic rate of U138/TMZ and LN18/TMZ cells was increased by si-circ_0005198-2, and partially reversed through co-transfection with miR-198 inhibitor (Figure 5E). Likewise, knockdown of circ_0005198 decreased the IC50 value of cells, and this effect was partially abolished by the silencing of miR-198 (Figure 5F). These results suggested that circ_0005198 regulated TMZ resistance in glioma via negatively regulating miR-198 expression in U138/TMZ and LN18/TMZ cells.

### TRIM14 was a target gene of miR-198

**Figure 6:**
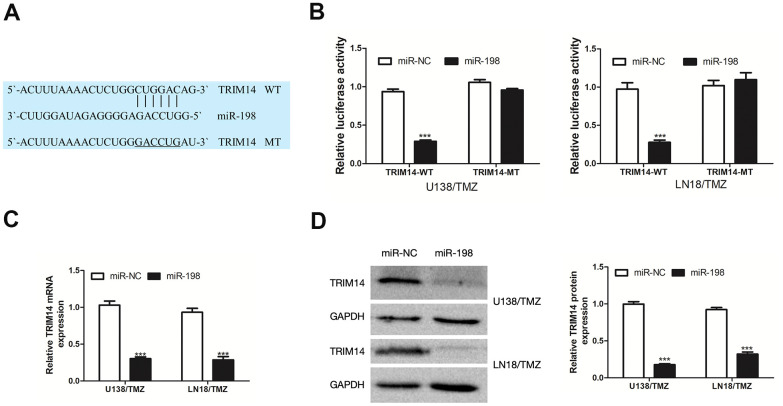
**TRIM14 was a target gene of miR-198.** (**A**) The predicted target region of TRIM14 3’UTR with the miR-198 binding sites and mutant binding sites was shown. (**B**) Dual-luciferase reporter assay was used to detect the interaction between miR-198 and TRIM14 in U138/TMZ and LN18/TMZ cells. (**C, D**) qRT-PCR and WB analysis were used to measure the effect of miR-198 expression on the mRNA and protein expression of TRIM14. ***P< 0.001.

To investigate the molecular mechanism of circ_0005198 in TMZ-resistant glioma cells, the website database TargetScan was adopted for predicting the target gene of miR-198. A putative miR-198 binding site was identified in the 3’UTR of TRIM14 (Figure 6A). As indicated from the results of dual-luciferase reporter assay, miR-198 over-expression considerably suppressed the luciferase activity of TRIM14-WT in U138/TMZ and LN18/TMZ cells, whereas TRIM14-MT was not affected (Figure 6B). By ascertaining the mRNA and protein expression, we reported that over- expression of miR-198 hindered TRIM14 expression in U138/TMZ and LN18/TMZ cells (Figure 6C, 6D). These results indicated that TRIM14 was the putative target of miR-198.

### Silencing of TRIM14 inhibited the progression of TMZ-resistant glioma cells

**Figure 7:**
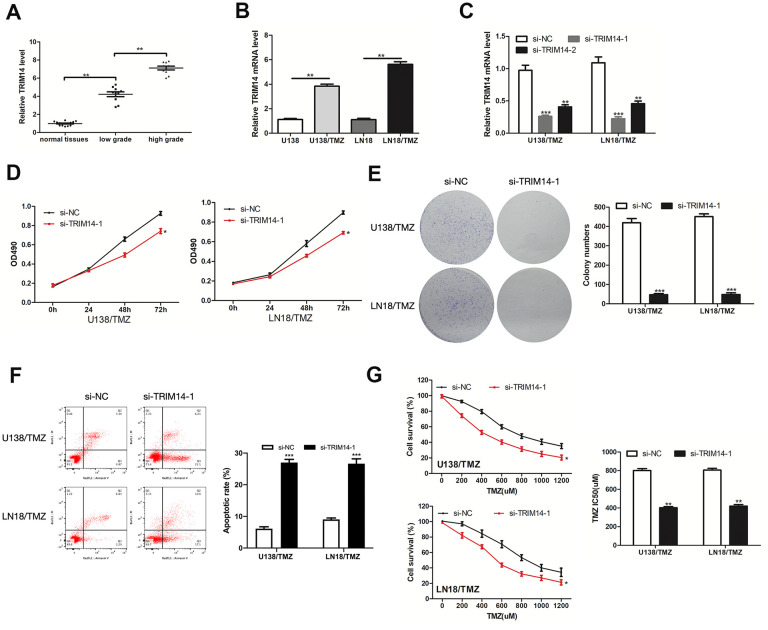
**TRIM14 was up-expressed in glioma and its silencing inhibited the progression of TMZ-resistant glioma cells.** (**A, B**) Analysis of the relative expression of TRIM14 in glioma tissues, glioma cells (U138 and LN18) and TMZ-resistant glioma cells (U138/TMZ and LN18/TMZ). (**C**) qRT-PCR was used to measure the expression of TRIM14 in U138/TMZ and LN18/TMZ cells transfected with si-TRIM14. (**D, E**) The viability was detected by CCK-8 and colony formation assays in transfected cells. **(F)** Apoptotic rate was detected by flow cytometry. (**G**) The TMZ resistance of cells was measured by CCK-8 assay. *P < 0.05, **P < 0.01, ***P < 0.001.

We investigated the impacts exerted by TRIM14 on TMZ resistance of glioma. TRIM14 was highly expressed in glioma tissues and TMZ-resistant glioma cells (Figure 7A, 7B). Then we knocked down TRIM14 expression in U138/TMZ and LN18/TMZ cells (Figure 7C). CCK-8 and colony formation assays revealed that inhibition of TRIM14 reduced U138/TMZ and LN18/TMZ cells proliferation (Figure 7D, 7E). The apoptotic rate of U138/TMZ and LN18/TMZ cells transfected with si-TRIM14-1 was increased (Figure 7F). The IC50 value of cells was significantly decreased after TRIM14 knockdown, indicating that TMZ resistance was suppressed (Figure 7G). All the above data determined that TRIM14 expression was crucial to the occurrence of TMZ resistance of glioma cells.

### MiR-198 mediated the regulatory effects of TRIM14 on the progression of TMZ-resistant glioma cells

**Figure 8:**
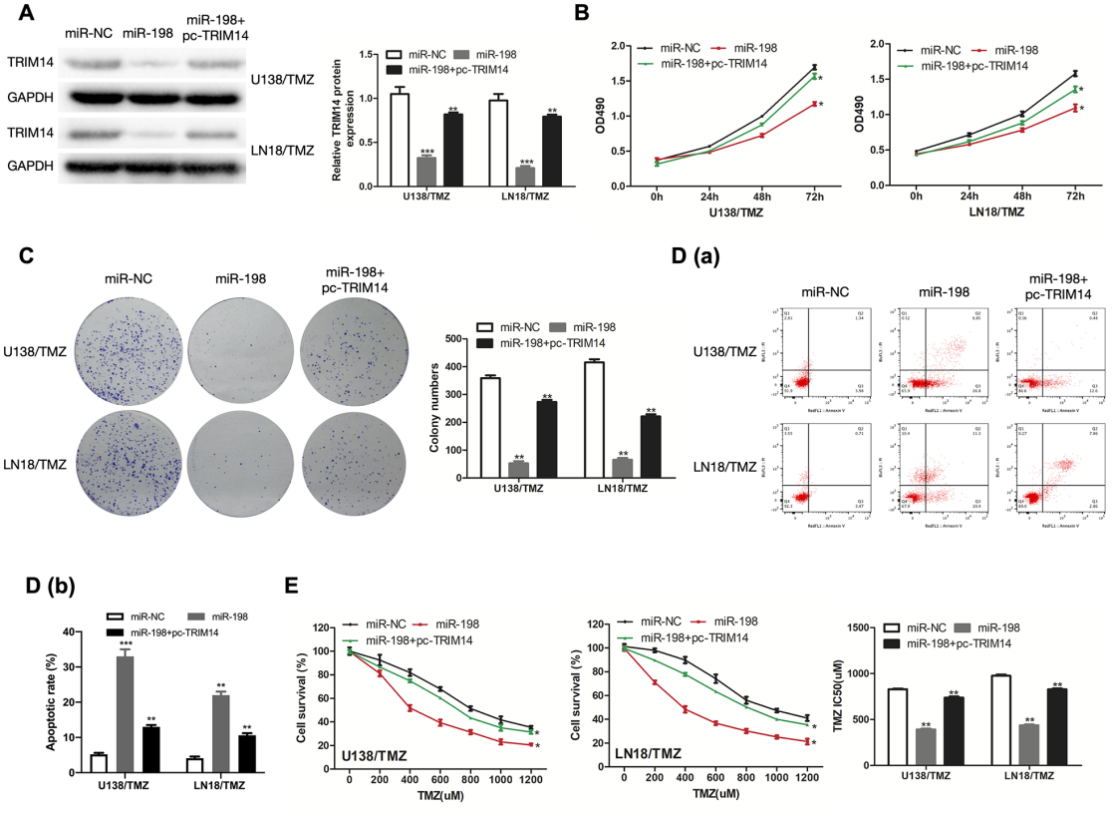
**MiR-198 mediated the regulatory effects of TRIM14 on the progression of TMZ-resistant glioma cells.** (**A**) Western Blotting analysis was used to measure TRIM14 expression in U138/TMZ and LN18/TMZ cells after co-transfected with miR-198 mimic and TRIM14 overexpression plasmid (pc-TRIM14). (**B**, **C**) The viability of U138/TMZ and LN18/TMZ cells co-transfected with miR-198 mimic and pc-TRIM14 was detected by CCK-8 and colony formation assays. (**D**) Apoptotic rate was detected by flow cytometry. (**E**) The TMZ resistance of cells was measured by CCK-8 assay. *P < 0.05, **P < 0.01, ***P < 0.001.

**Figure 9:**
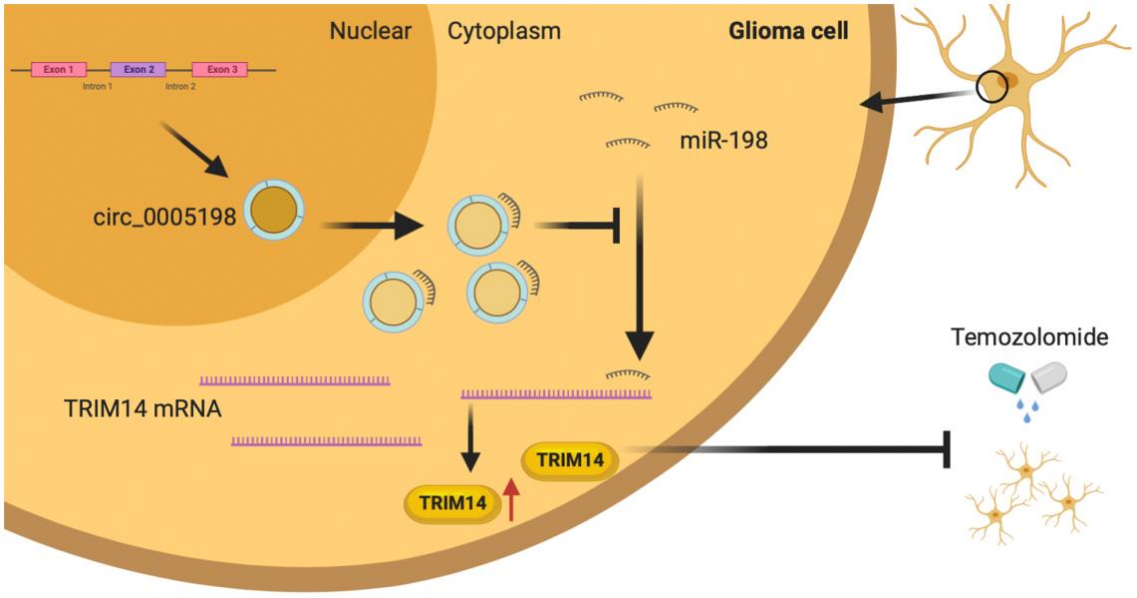
**Schematic diagram.** Circ_0005198 acted as a sponge of miR-198 to enhances temozolomide resistance by regulating downstream TRIM14 expression in glioma cells.

To determine the regulatory relationship between TRIM14 and miR-198, we measured the protein expression of TRIM14 in U138/TMZ and LN18/TMZ cells transfected miR-198 mimic and TRIM14 over-expression plasmid (pc-TRIM14). The results indicated that miR-198 over-expression inhibited the expression of TRIM14, while the over-expression of TRIM14 could increase its expression (Figure 8A). CCK-8 and colony formation assays showed that the proliferation inhibition mediated by miR-198 mimic can be partially reversed by co-transfection with TRIM14 over-expression plasmid (Figure 8B, 8C), demonstrating that miR-198 inhibited cell proliferation by restricting TRIM14 expression. Moreover, flow cytometry assay indicated that the apoptotic rate was up-regulated by miR-198 mimic and partially reversed through co-transfection with pc- TRIM14 (Figure 8D). Likewise, miR-198 mimic lowered the IC50 value of cells; such effect was partially abolished by overexpressing TRIM14 via the transfection of pc-TRIM14 (Figure 8E). Our results suggested that TRIM14 expression was altered by miR-198 on the progression of TMZ-resistant glioma cells. Taken together, our study revealed that circ_0005198 exerted its function as a ceRNA through sponging miR-198 to regulate TRIM14 expression, and therefore contributed to TMZ resistance in glioma (Figure 9).

## DISCUSSION

Glioma refers to an aggressive tumor arising from glial cells of the central nervous system. Despite the development of new treatment options, the survival rate of glioma remains low. In particular, glioblastomas patients have a median survival of 15 months [21]. Surgery and the following radiotherapy assisted with temozolomide (TMZ) or cisplatin adjuvant chemotherapy is the normal strategy to treat glioma; however, the prognosis remains poor as there is growing resistance to the chemotherapeutic drugs [22, 23]. For this reason, researches stressing the molecular and cellular mechanisms regarding the glioma chemoresistance would prompt the development of more effective treatments on the malignant glioma.

Currently, proteins, non-coding RNAs and many other molecules have been identified as the potential biomarkers to diagnose or prognostically predict glioma [24–27]. Growing evidence has proved the crucial function of circRNAs to tumor occurrences and progressions [28, 29]. Owing to its naturally closed loop structure, circRNA can be stable in the tissues and serum [7]. So far, there have been many studies focusing on circRNAs regulating the progression of glioma. For instance, Chao Yi et al. showed that upregulation of circ_0034642 demonstrated a poor glioma prognosis and facilitated the proliferation and invasion via the miR-1205/BATF3 axis [30]. Fei Shi et al. reported that circ_0014359 contributed to the glioma progression via sponging miR-153 and regulating PI3K signal [31]. In existing studies, circ_0005198 was correlated with glioma development [12]. In the present study, we showed that circ_0005198 expression was up-regulated in glioma tissues, serum and TMZ-resistant glioma cells. Silencing of circ_0005198 suppressed the resistance of glioma cells to TMZ, inhibited the proliferation and enhanced the apoptosis of TMZ-resistant glioma cells. These results confirmed the positive effect of circ_0005198 on glioma resistance and provided a theoretical basis for circ_0005198 to become an ideal biomarker for glioma resistance diagnosis and treatment.

Numerous studies have shown that miRNA regulatory networks played an essential effect on the progression and chemoresistance of cancer [32, 33]. Liu et al. showed that miR-497-5p enhanced apoptosis in OC cells [34]. Besides, Ueda et al. suggested that miR-27a ameliorated chemoresistance of breast cancer cells by disruption of reactive oxygen species homeostasis [35]. Based on the findings here, circ_0005198, as “miRNA sponge”, interacted with the miR-198 and suppressed the miR-198 activity. As reported in this study, miR-198 could hinder the TMZ resistance and suppress the progression of TMZ-resistant glioma cells, complying with the conclusion of Nie et al. [36]. Furthermore, we found that the effect of circ_0005198 on the progression of TMZ-resistant glioma cells could be achieved by silencing of miR-198. For this reason, the mentioned results uncovered that circ_0005198 regulated glioma resistance through the inhibitory effect on miR-198. Meanwhile, we showed that TRIM14 as a target of miR-198 and contributed to TMZ resistance.

Investigations have been reported on the involvement of TRIM14 in glioma chemoresistance. Tan et al. found that TRIM14 promoted chemoresistance in gliomas by activating Wnt/β-catenin signaling pathway [20]. Our study verified that TRIM14 was highly expressed in glioma and its silencing inhibited the progression of TMZ-resistant glioma cells. We showed that TRIM14 expression was required to maintain TMZ resistance in glioma cells. Furthermore, TRIM14 over-expression could invert the inhibiting effect of miR-198 on the progression of TMZ-resistant glioma cells, thereby confirming that TRIM14 functioned as a target gene of miR-198.

Although there are considerable reports on circRNAs, more potential functions of circRNAs in cancer require further investigation. This study demonstrated that circ_0005198 induced TMZ resistance of glioma by regulating the miR-198-TRIM14 axis, and this novel molecular mechanism added a new respective to the oncogenic function of circ_0005198 in glioma.

## MATERIALS AND METHODS

### Clinical samples

Twenty glioma samples were collected from patients receiving surgical resection and 15 normal brain tissues were acquired from individuals died in traffic accidents from January 2016 to December 2019 in the Department of Neurosurgery at the Third Xiangya Hospital of Central South University. Glioma specimens were diagnosed pathologically and split to low-grade group (stage I-II, n=10) and high-grade group (stage III-IV, n=10) abiding by the WHO classification system by at least 2 advanced clinical pathologists. Prior to surgery, none of these patients was treated by chemotherapy or radiotherapy. Besides, we collected blood samples from these patients. The serum extracted from the blood samples were collected using standard procedures. The study was conducted in accordance with the Declaration of Helsinki. Written informed consents were collected from patients or their relatives regarding the utilization of their specimens and disease information. The project was approved by the Third Xiangya Hospital of Central South University. All samples were stored in the liquid nitrogen following resection till use.

### Cell culture

The human glioma cell lines U87, U251, U138 and LN18, as well as normal human astrocytes (NHA) were provided by ATCC. U138 and LN18 were administrated with rising concentrations of TMZ to form TMZ-resistant glioma cells (U138/TMZ and LN18/TMZ). All cells were kept and incubated in Dulbecco’s Modified Eagle Medium (DMEM; Hyclone, South-Logan, UT, USA) added with 10% fetal bovine serum (FBS; Hyclone) and without antibiotics. Cells received the incubating process in wet atmosphere containing 5% CO_2_ at 37° C.

### Quantitative real-time polymerase chain reaction (qRT-PCR)

Total RNAs were harvested from tissues, serum or cells with the use of Trizol reagent (Invitrogen, Carlsbad, CA, USA) according to the instructions of manufacturer. For circRNA and mRNA, PrimeScript™ RT reagent Kit (TaKaRa, Dalian, China) helped to generate the cDNA; TB Green™Premix Ex Taq™ II (TaKaRa) helped to perform the real-time PCR; GAPDH was applied as the endogenous control. In terms of miRNA, miRcute Plus miRNA First-Strand cDNA Kit (TIANGEN) was adopted for generating cDNA; miRcute Plus miRNA qPCR Kit (SYBR Green) was adopted to perform real-time PCR to measure the comparative expression of circ_0005198, miR-198 and TRIM14 with 2^−ΔΔCt^ methods. U6 was considered internal controls. Sangon Biotech (Shanghai, China) contributed to synthesizing primers. Primer sequences were: circ_0005198, forward: 5’-GGTTGCACTAGCGTTATTC-3’ and reverse: 5’-CAACATCCATTGGGTCTCC-3’; TRIM14, forward: 5’-GCAGAAACTCAGCCAAGAA-3’ and reverse: 5’-CTTGACTCTGCATTAGCCT-3’; GAPDH, forward: 5’-GTCTCCTCTGACTTCAACAGCG-3’ and reverse: 5’-ACCACCCTGTTGCTGTAGCCAA-3’; U6, forward: 5’-CTCGCTTCGGCAGCACA-3’ and reverse: 5’-AACGCTTCACGAATTTGCGT-3’; miR-198 forward: 5’-GGUCCAGAGGGGAGAUAGGUUC-3’. Reverse primer for the miR-198 was provided by the miRcute Plus miRNA qPCR Kit (SYBR Green).

### Cell transfection

Circ_0005198 small interfering RNA (si-circ_0005198) or their negative controls (si-NC), TRIM14 small interfering RNA and over-expression plasmid (si-TRIM14 and pc-TRIM14) or their negative controls (si-NC and pc-NC) were provided by RiboBio (Guangzhou, China). Genepharma Company (Shanghai, China) contributed to synthesizing miR-198 mimic and inhibitor or their negative controls (miR-NC and anti-miR-NC). Lipofectamine^TM^3000 transfection reagent (Invitrogen, Carlsbad, CA, USA) was employed for building cells transfecting process.

### Cell TMZ resistance and proliferation assays

For determining the TMZ resistance and proliferation of cells, CCK-8 reagent (Dojindo, Japan) was used to detect cell viability. U138/TMZ and LN18/TMZ cells underwent the seeding process into 96-well plates. The cells were then treated with TMZ at a range of levels (0 μM, 200 μM, 400 μM, 600 μM, 800 μM, 1000 μM and 1200 μM) for 48 h. CCK-8 reagent was added to respective well and then incubated in the incubator containing 5% CO2 at 37° C for 2 h. For evaluating the TMZ resistance of cells, we measured the absorbance at 490 nm and then calculated the half-maximal inhibitory concentration (IC50). Furthermore, U138/TMZ and LN18/TMZ cells were administrated with CCK-8 at the specified time point after transfection. Then, we examined the absorbance at 490 nm for assessing cell proliferation.

### Colony formation assay

We seeded cells undergone transfection in six-well plates with culture medium containing 10% FBS and incubated throughout the night. Fourteen days later, methanol was adopted for fixing cells followed by staining treatment with 0.1% Crystal Violet. Under a light microscope, we counted the colonies.

### Flow cytometry

U138/TMZ and LN18/TMZ cells were gained after they were transfected for 48 h and stained with the Annexin V-fluorescein isothiocyanate (FITC)/Propidium iodide (PI) Apoptosis Detection Kit (Yeasen, Shanghai, China). For gaining fluorescence signals and ascertaining the apoptosis rate of U138/TMZ and LN18/TMZ cells, Flow cytometer (Thermo Fisher Scientific, Waltham, MA, USA) was employed.

### RNAs isolation from nucleus and cytoplasmic fractions

The PARIS™ Kit (Invitrogen) was applied to isolate the cytoplasmic fraction and nuclear fraction following the manufacturer’s directives. Briefly, we first harvested cells, followed by using cell fractionation buffer to lyse the harvested cells, and then separated cytoplasmic fraction and nuclear fraction with centrifugation. We also harvested supernatant containing cytoplasmic fraction and then transferred them to a fresh tube without RNase. The Cell Disruption Buffer was employed for lysing nuclear pellet. We mixed the nuclear lysate and cytoplasmic fraction with the 2X Lysis/Binding Solution and subsequently mixed them with 100% ethanol. A Filter Cartridge contributed to drawing sample mixture. Besides, Washing Solution was adopted for sample washing. The Elution Solution was employed for eluting the RNAs of cytoplasmic fraction and nuclear fraction. U6 snRNA and GAPDH acted as the positive control for the nuclear fraction and the cytoplasmic fraction, respectively.

### Dual-Luciferase reporter assay

The putative miR-198 binding sites were assessed in circ_0005198/TRIM14 3′-UTR. The pMIR-REPORT™ (RiboBio), covering wild type (WT) or mutant (MT) circ_0005198/TRIM14 3′-UTR sequences was employed for carrying out the dual-luciferase reporter assay. Cells (1×10^5^) saw transient co-transfection with the miR-198 mimic or the negative control accompanied by WT or MT circ_0005198/TRIM14 3′-UTR vector. 48h later, we harvested the cells. The luciferase results were detected with the Luc-Pair™ Duo-Luciferase Assay Kit (Yeasen).

### RNA immunoprecipitation (RIP) assay

A Magna RNA-Binding Protein Immunoprecipitation Kit (Millipore, USA) contributed to implementing an RNA immunoprecipitation. In brief, a RIP buffer covering magnetic beads was employed for incubating entire-cell lysate, displaying conjugation with human anti-Ago2 antibody (1:50, Millipore) or normal mouse IgG (Millipore), classified to be a negative control. Proteinase K buffer helped to incubate samples. The qRT-PCR contributed to extracting and analyzing immunoprecipitated RNA to reveal the presence of circ_0005198 and miR-198 expression.

### Western blot (WB) analysis

BCA protein assay kit (Sigma, USA) was applied to extracting the total proteins derived from glioma cells and detect protein concentration. Proteins were resolved with 10% sodium dodecyl sulfate/polyacrylamide gel electrophoresis (SDS/PAGE; Sigma). After separation, the proteins were transferred to the PVDF membrane (Bio-Rad, USA). The membranes were incubated with TRIM14 antibody (Cell Signaling Technology, USA), and GAPDH (Santa Cruz Biotechnology, USA) at 4° C overnight. Afterwards, blotted membranes underwent 2h of incubation with HRP-conjugated secondary antibody at ambient temperature. ECL Substrates (Millipore) contributed to visualizing the signals.

### Statistical analysis

SPSS 21.0 software was applied in statistical analysis. The experimental processes in the study were carried out in triplicate, and Mean ± SD represents the results. The two-tail Student’s t-test or one-way ANOVA was applied for assessing the difference between the two groups. Correlations were analyzed by Spearman rank correlation. It was set that the difference was of statistical significance with P value < 0.05.
